# Widely Targeted Metabolomics Analysis to Reveal Metabolite of *Morus alba* L. in Different Medicinal Parts

**DOI:** 10.3390/molecules29173981

**Published:** 2024-08-23

**Authors:** Xinwei Wang, Yiyun Qian, Min Wei

**Affiliations:** Jiangsu Key Laboratory for the Research and Utilization of Plant Resources, Institute of Botany, Jiangsu Province and Chinese Academy of Sciences, Nanjing 210014, China; wangxw.9@foxmail.com (X.W.); qianyiyun@hotmail.com (Y.Q.)

**Keywords:** *Morus alba* L., widely targeted metabolites, chemical composition, properties of Chinese medicines

## Abstract

*Morus alba* L. is a tradition medical and edible plant. It is rich in many important bioactive components. However, there is a dearth of systematic information about the components. Here, the Mori Cortex, Mori Folium, Mori Fructus, and Mori Ramulus were studied. Ultrahigh-performance liquid chromatography-mass spectrometry (UHPLC–MS) is used to study primary and secondary metabolites. Eight hundred two metabolites were identified and classified into 10 different categories in total. Correlation analysis, hierarchical clustering analysis, and principal component analysis of metabolites showed that different parts of the sample could be significantly different. In different medicinal parts, alkaloids accounted for 4.0%, 3.6%, 5.1%, and 4.5%; flavonoids accounted for 0.7%, 27.2%, 5.6%, 1.2%; terpenes accounted for 20.1%, 2.1%, 2.6%, 2.5%. Furthermore, the abundance of phenols, phenylpropanoids, and lipids metabolites sequentially accounted for 2.3–4.4%, 0.5–1.8%, and 2.4–5.3%. These results have improved our understanding of metabolites and provided a reference for research on the medicinal and edible value of Morus alba L. In addition, the study reveals the correlation between the components of Traditional Chinese medicine and the basic theory of TCM properties and reinterprets the ancient wisdom in the world’s traditional herbs through the perspective of modern science.

## 1. Introduction

*Morus alba* L. is a perennial plant from the Moraceae family, which has been cultivated elsewhere, such as in Asia, Europe, America, and Africa [1]. In China, *Morus alba* L. has a planting history of 5000 years and is widely planted in many provinces. The Chinese Pharmacopoeia 2020 edition includes Mori Cortex Radices (*Morus alba* root cortex, MC), Mori Folium (*Morus alba* leaf, MFo), Mori Fructus (*Morus alba* fruit, MFr), and Mori Ramulus (*Morus alba* dried twigs, MR) from the *Morus alba* L. in different medicinal parts.

In Traditional Chinese Medicine theory, MFos are known for their effects on dispelling wind, clearing heat, moistening the lungs, and benefiting the throat. They also function in clearing the liver and improving eyesight. MRs are effective in expelling wind dampness and benefiting the joints. MC has the properties of clearing phlegm from the lungs, relieving asthma, and reducing swelling. MFo is used to nourish yin, enrich the blood, promote saliva production, and moisten dryness.

Modern biomedical research indicates that the mulberry plant contains a variety of chemically active constituents. These bioactive substances are responsible for the mulberry’s pharmacological effects and high medicinal value. To date, the chemical components isolated and identified from different medicinal parts of the mulberry tree primarily include flavonoids, alkaloids, polysaccharides, phenolic acids, coumarins, terpenoids, stilbenes, amino acids, and numerous other compounds [2,3].

*Morus alba* L. is rich in natural active substances, including alkaloids, phenols, flavonoids, amino acids, and terpenoids [1,4,5,6,7]. These different medicinal parts and bioactive phytochemicals from *Morus alba* L. deserve further analysis in activity. Traditional Chinese medicine has different applications for different parts of the mulberry tree, and modern research has found that different parts have different curative effects [3,8].

The study reveals the correlation between the components of Traditional Chinese medicine and the basic theory of TCM properties and reinterprets and it uses ancient wisdom in the world’s traditional herbs from the perspective of modern science. Metabolomics can be used to analyze the variation of metabolite components fully, which have been widely used in medicine development and efficacy evaluation and offer insights into molecular mechanisms in biological samples [5,6,7,8,9,10]. Metabolomics uses a variety of tools, including high-performance liquid chromatography, mass spectrometry, and nuclear magnetic resonance spectroscopy [11]. Ultra-high-performance liquid chromatography coupled with mass spectrometry (UHPLC–MS) is a leading technology for detecting plant metabolites, providing an important tool for systematic analysis of TCM metabolites [12]. We conducted a comprehensive metabolite analysis of each part to further elucidate their distinct efficacies. In the present study, MFr, MFo, MR, and MC of *Morus alba* L. were collected, and untargeted metabolomics analysis was carried out by UHPLC–MS. Furthermore, the accumulation rules and activity orientation of different metabolites in different parts of *Morus alba* L. were evaluated in order to provide a reference for the rational utilization of *Morus alba* L. resources.

## 2. Results

### 2.1. Metabolic Profiling Based on UHPLC–MS Analysis

**Figure 1:**
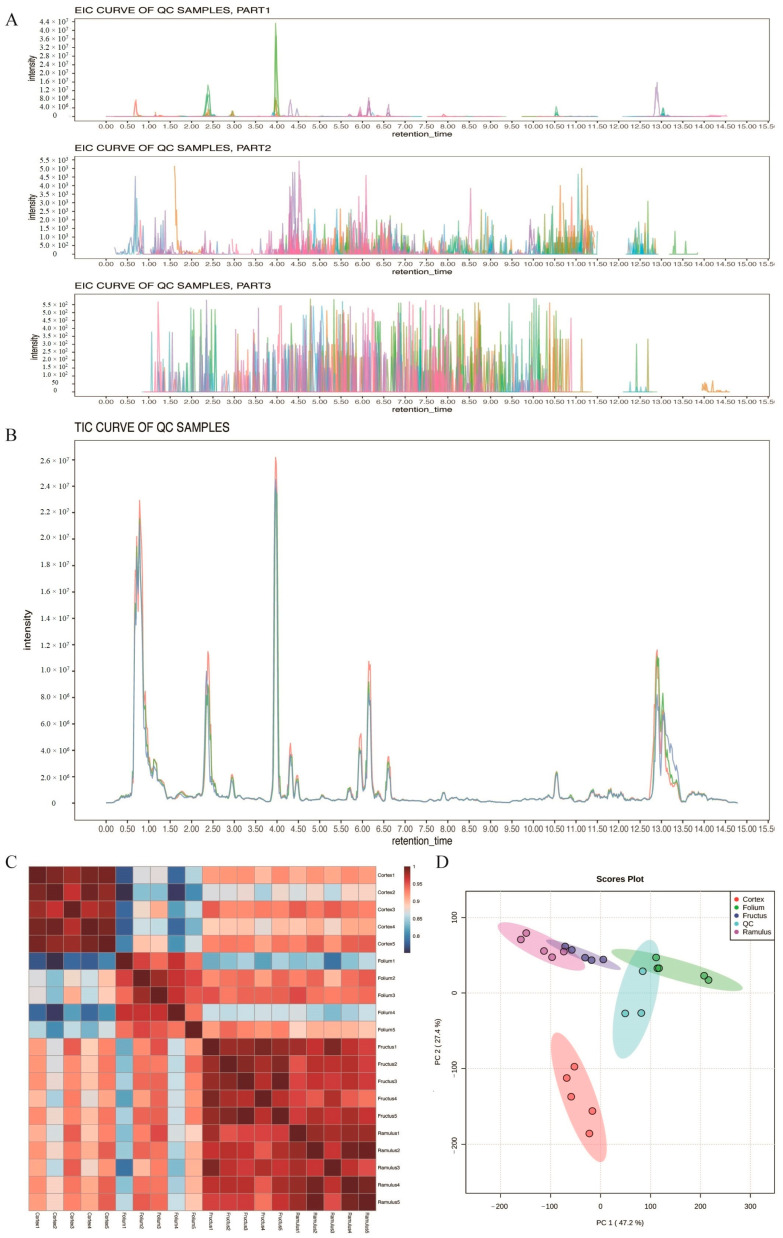
All metabolites quality control analysis. (**A**) Extracted ion chromatogram (EIC) of QC sample. (**B**) PCA diagram of all samples. (**C**) Total ions current (TIC) of QC sample. (**D**) Pearson’s correlation coefficients of all samples.

In order to investigate the chemical composition of different medicinal parts, including Mori Folium, Mori Cortex, Mori Ramulus, and Mori Fructus, the metabolites were identified by UHPLC–MS analysis. All compounds exhibited symmetrical chromatographic peaks and achieved good chromatographic separation of each target compound in the extracted ion chromatogram and total ions (Figure 1A,B). There was a significant correlation among biological replicates in different medicinal parts (Figure 1C). In order to further analyze the degree of variation between samples, PCA was performed on the metabolite spectra of the four samples PCA (Figure 1D). In summary, these results indicate significant differences in the metabolic characteristics of different medicinal parts.

**Figure 2:**
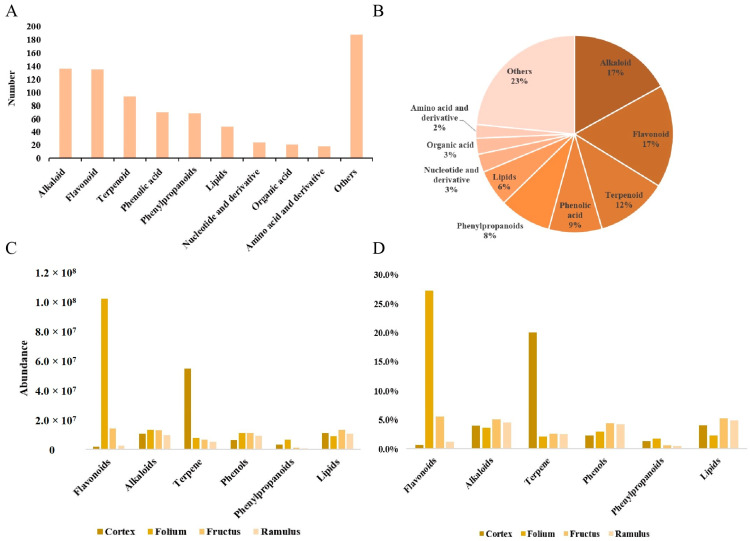
UHPLC–MS analysis of metabolites in *Morus alba* L. (**A**) Types and number of identified metabolites. (**B**) Types and proportions of the identified metabolites. (**C**) The ratio of metabolite abundance in different medicinal parts. (**D**) The abundance of metabolites in different medicinal parts.

A total of 802 metabolites were identified, including 123 primary metabolites and 679 secondary metabolites. Meanwhile, metabolites could be classified into alkaloids (136, 17%), flavonoids (135, 17%), terpenoids (94, 12%), phenolic acid (70, 9%), phenylpropanoids (68, 8%), lipids (48, 6%), nucleotide and derivative (24, 3%), organic acid (21, 3%), amino acid and derivate (18, 2%), others (188, 23%) (Figure 2A,B). From the figure it can be seen that alkaloids, flavonoids, phenolic acids, and terpenes are the main metabolites.

The total abundance of metabolites of MC was 269,675,586, MFo was 371,346,215, MFr was 255,244,012, and MR was 219,446,313. The top six most abundant metabolites were alkaloids, flavonoids, terpenes, phenols, phenylpropanoids, and lipids. Among the different medicinal parts (MC, MFo, MFr, and MR), respectively, alkaloids accounted for 4.0%, 3.6%, 5.1%, and 4.5%, flavonoids accounted for 0.7%, 27.2%, 5.6%, 1.2%, terpene accounted for 20.1%, 2.1%, 2.6%, 2.5%. Furthermore, the abundance of phenols, phenylpropanoids, and lipid metabolites sequentially accounted for 2.3–4.4%, 0.5–1.8%, and 2.4–5.3% (Figure 2C,D). Particularly, the abundance of flavonoids in MFo was 55-fold, 7-fold, and 37-fold that of MC, MFr, and MR. For the moment, terpenes in MC were 7-fold, 8-fold, and 10-fold that of MFo, MFr, and MR.

The top 10 most abundant metabolites of MC were morroniside, D-alpha-aminobutyric acid, adenosine, 4-hydroxyphenylacetylglutamic acid, piperlonguminine, lubiprostone, p-octopamine, nandrlone, esculin, and miltirone. In the interim, the top 10 most abundant metabolites of MFo were isoquercitrin, adenosine, astragalin, D-alpha-aminobutyric acid, rutin, 5’-S-methyl-5’-thioadenosine, 4-hydroxyphenylacetylglutamic acid, 2-phenylacetamide, p-octopamine, and piperlonguminine. For them, the top 10 most abundant metabolites of the MFr were D-alpha-aminobutyric acid, 4-hydroxyphenylacetylglutamic acid, piperlonguminine, p-octopamine, rutin, lubiprostone, adenosine, nandrolone, miltirone and isoquercitrin. At the same time, the top 10 most abundant metabolites of MR were adenosine, D-alpha-aminobutyric acid, 4-hydroxyphenylacetylglutamic acid, p-octopamine, lubiprostone, piperlonguminine, 2-phenylacetamide, nandrolone, miltirone, and oleic acid. On this basis, adenosine, D-alpha-Aminobutyric acid, 4-hydroxyphenylacetylglutamic acid, p-Octopamine, and piperlonguminine aslo were highly abundant, when compared with each other in these four parts. The contents of monoside and esculin in MC were high, the contents of isoquercitrin and astragalin in MFo were high, and the contents of oleic acid in MR were high (Table S1)

### 2.2. Differential Metabolites Analysis

**Figure 3:**
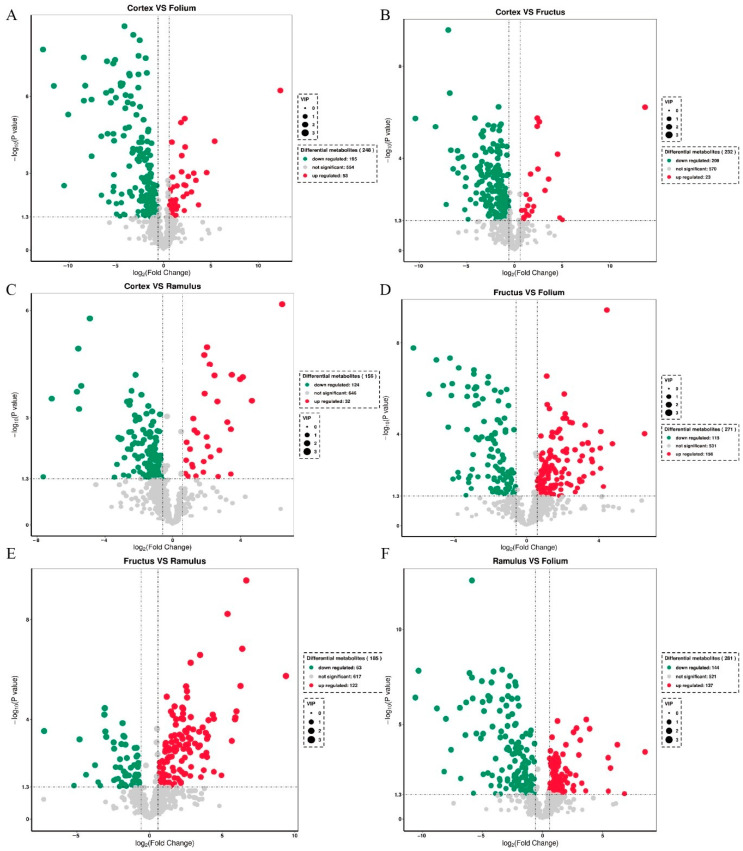
Volcano plot of the differential metabolites. (**A**) MC vs. MFo. (**B**) MC vs. MFr. (**C**) MC vs. MR. (**D**) MFr vs. MFo. (**E**) MFr vs. MR. (**F**) MR vs. MFo.

To identify differentially accumulated metabolites in different medicinal parts, the absolute value of log fold change (FC) ≥ 0.6, *p*-value 0.05, and variable importance (VIP) ≥ 1 were used as the screening criteria. A total of 248 differentially accumulated metabolites (DAMs) were detected between MC and MFo, including 60 flavonoids, 33 phenylpropanoids, and 30 terpene metabolites (Figure 3A). A total of 232 DAMs were detected between MC and MFr, including 48 flavonoids, 34 alkaloids, 21 terpenes, and 21 phenylpropanoids (Figure 3B). 156 DAMs were detected between MC and MR, including 33 flavonoids, 24 phenylpropanoids, and 22 alkaloids (Figure 3C). A total of 271 DAMs were detected between MFr and MFo, including 42 flavonoids, 33 terpenes, and 33 alkaloids (Figure 3D). 185 DAMs were detected between MFr and MR, including 41 flavonoids, 28 alkaloids, and 20 terpenes (Figure 3E). A total of 281 DAMs were detected between MR and MFo, including 57 flavonoids, 40 alkaloids, and 37 phenylpropanoids (Figure 3F).

**Figure 4:**
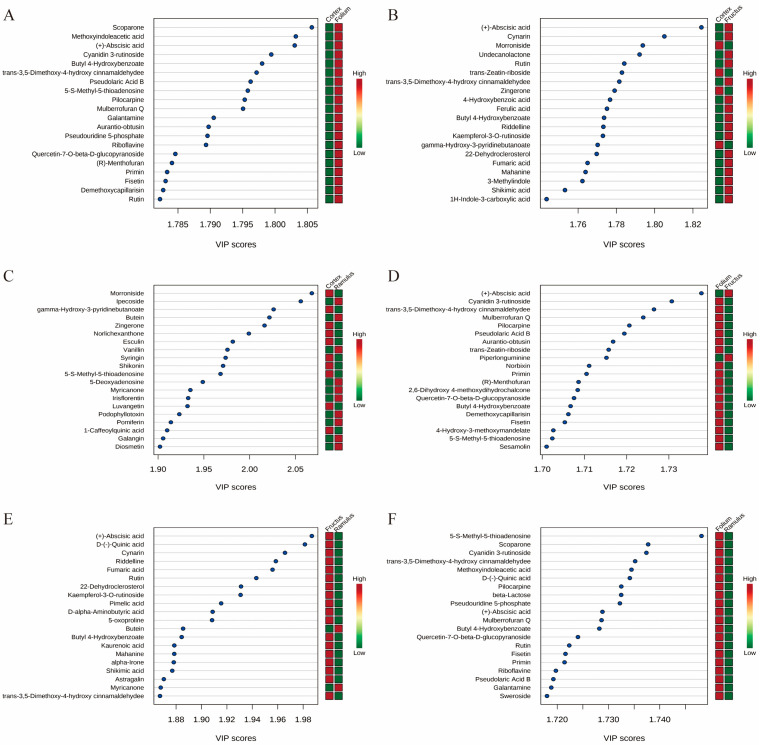
VIP scatter plot of the differential metabolites. (**A**) MC vs. MFo. (**B**) MC vs. MFr. (**C**) MC vs. MR. (**D**) MFr vs. MFo. (**E**) MFr vs. MR. (**F**) MR vs. MFo.

**Figure 5:**
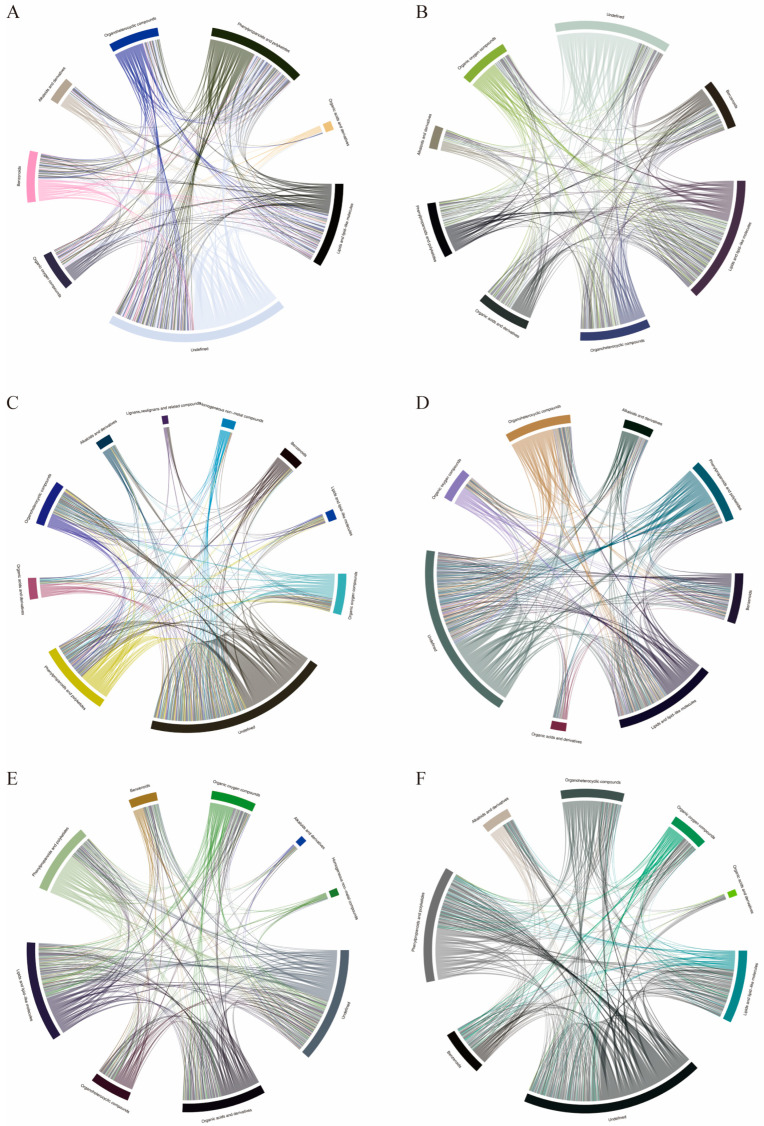
Chord diagrams of the differential metabolites. (**A**) MC vs. MFo. (**B**) MC vs. MFr. (**C**) MC vs. MR. (**D**) MFr vs. MFo. (**E**) MFr vs. MR. (**F**) MR vs. MFo.

**Figure 6:**
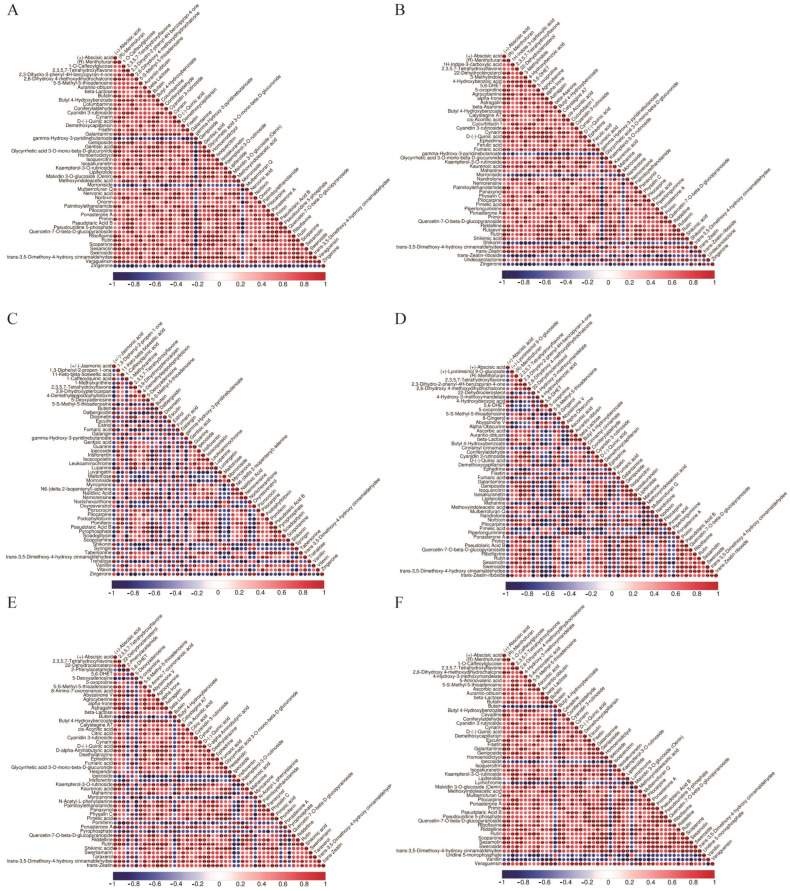
Correlation heatmap of the differential metabolites. (**A**) MC vs. MFo. (**B**) MC vs. MFr. (**C**) MC vs. MR. (**D**) MFr vs. MFo. (**E**) MFr vs. MR. (**F**) MR vs. MFo.

**Figure 7:**
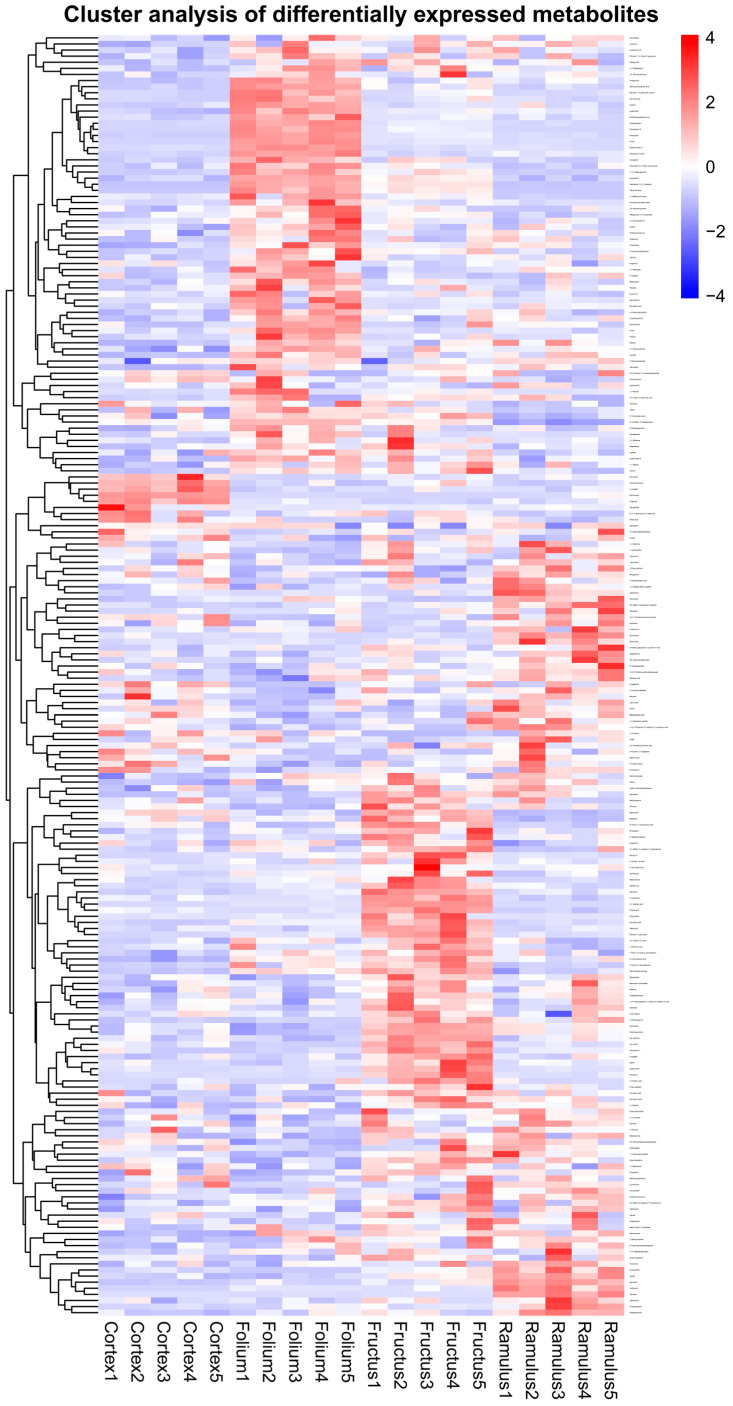
Correlation heatmap and cluster analysis of the differential metabolites.

Differential metabolite VIPs are visualized in scattered form, showing differential metabolites in the top 20 VIPs (Figure 4). In order to reveal the co-regulatory relationship between various metabolites more intuitively, we use differential metabolite chord diagrams (Figure 5). The correlation heatmap shows the correlation between the differential metabolites (Figure 6). Finally, a clustering heatmap of all differential metabolites is shown for all samples (Figure 7). The results revealed that these 802 compounds had different contents in the different medicinal samples from *Morus alba* L.

### 2.3. KEGG Pathway Enrichment Analysis

We mapped the metabolites from the MC, MFo, MFr, and MR to the KEGG database. Metabolism was the largest category of these pathways. Only a few pathways were classified in the environmental information processing category. A total of 248 differential metabolites were enriched in the 56 pathways between MC and MFr. In total, 232 differential metabolites were enriched in 63 pathways between MC and MFo. A total of 156 differential metabolites were enriched in 42 pathways between MC and MR. A total of 271 differential metabolites were enriched in 68 pathways between MFr and MFo. A total of 185 differential metabolites were enriched in 52 pathways between MFr and MR., and 281 differential metabolites were enriched in 65 pathways between MR and MFo. The top enriched KEGG pathways were mainly involved in flavonoid biosynthesis, nucleotide metabolism, amino acid biosynthesis, phenylpropene biosynthesis, and others.

**Figure 8:**
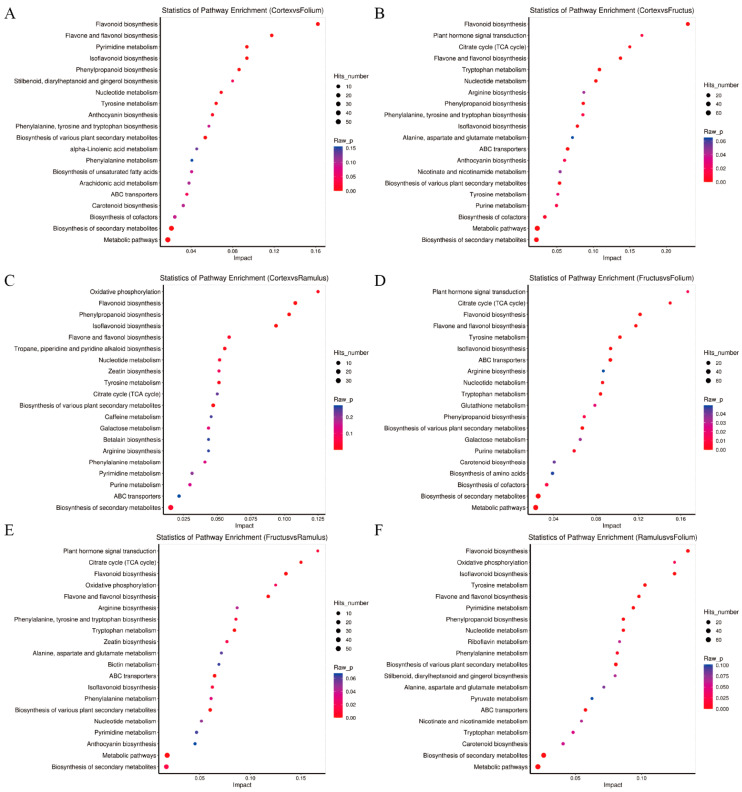
KEGG pathway enrichment analysis of the differential metabolites. (**A**) MC vs. MFo. (**B**) MC vs. MFr. (**C**) MC vs. MR. (**D**) MFr vs. MFo. (**E**) MFr vs. MR. (**F**) MR vs. MFo.

Further KEGG pathway enrichment analysis was conducted, and the 20 pathways with the highest enrichment levels are displayed on the bubble plot (Figure 8A–F). The comparison of MC vs. MFo revealed that flavonoid biosynthesis, flavone and flavanol biosynthesis, pyrimidine metabolism, isoflavonoid biosynthesis, phenylpropanoid biosynthesis, and tyrosine metabolism were the significantly enriched metabolic pathways. The comparison of MC vs. MFr revealed that ABC transporters, nucleotide metabolism, phenylpropanoid biosynthesis, isoflavonoid biosynthesis, citrate cycle (TCA cycle), and biosynthesis of cofactors. The comparison of MC vs. MR revealed that isoflavonoid biosynthesis was a significantly enriched metabolic pathway. The comparison of MFr vs. MFo revealed that ABC transporters, flavonoid biosynthesis, tyrosine metabolism, flavone and flavanol biosynthesis, tryptophan metabolism, isoflavonoid biosynthesis, nucleotide metabolism, citrate cycle, and purine metabolism were the significantly enriched metabolic pathways. The comparison of MFr vs. MR revealed that flavonoid biosynthesis, flavone and flavanol biosynthesis, tryptophan metabolism, ABC transporters, and citrate cycle were significantly enriched metabolic pathways. The comparison of MR vs. MFo revealed that flavonoid biosynthesis, isoflavonoid biosynthesis, tyrosine metabolism, pyrimidine metabolism, flavone and flavanol biosynthesis, ABC transporters, phenylpropanoid biosynthesis, nucleotide metabolism, and phenylalanine metabolism were the significantly enriched metabolic pathways.

**Figure 9:**
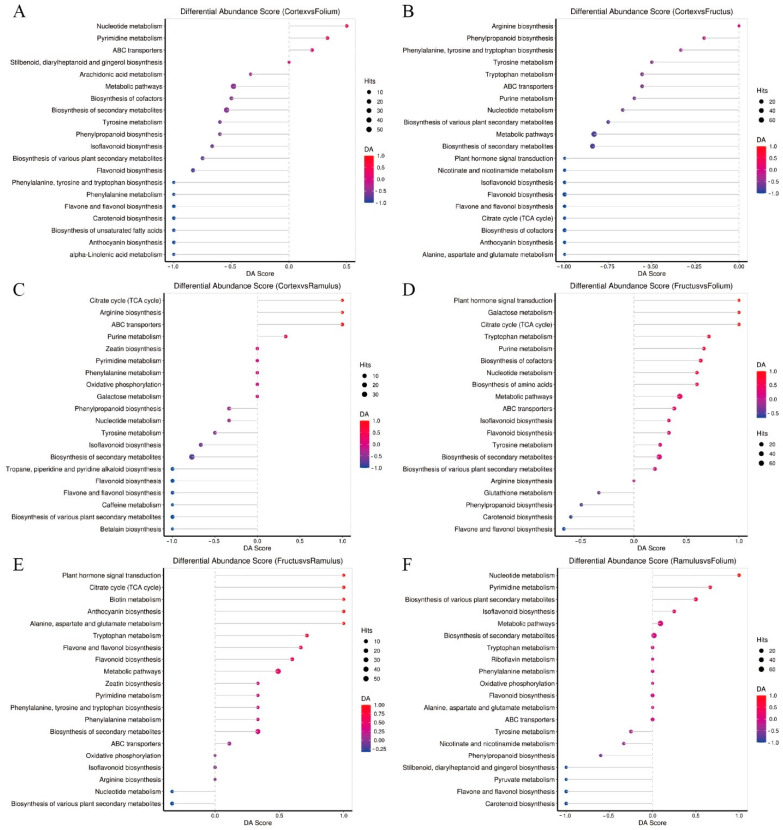
The differential abundance score plot of the significantly enriched metabolic pathway. (**A**) MC vs. MFo. (**B**) MC vs. MFr. (**C**) MC vs. MR. (**D**) MFr vs. MFo. (**E**) MFr vs. MR. (**F**) MR vs. MFo.

In the top 20 pathways, in common to each comparison group has ABC transporters, flavone and flavanol biosynthesis, flavonoid biosynthesis, isoflavonoid biosynthesis, and nucleotide metabolism significantly. Meanwhile, the differential abundance score plot of the significantly enriched metabolic pathway represents the overall expression of the pathway (Figure 9).

## 3. Discussion

*Morus alba* L., a traditional economic crop, has excellent biological properties and nutritional value. It is a medicinal and edible plant with abundant resources around the world [13,14,15,16,17]. Traditionally, various parts of *Morus alba* L., such as the roots, leaves, fruits, and branches, have been used as medicine for thousands of years; the roots and barks (Mori Cortex), leaves (Mori Folium), fruits (Mori Fructus), and branches (Mori Ramulus) of which are rich in essential active components [18]. This study systematically analyzed the metabolites of different medicinal parts of *Morus alba* L., providing a basis for its utilization.

The relative metabolite levels of different plant parts represent the characteristics and distribution of overall nutrition and phytochemicals, which helps to discover the most favorable plant parts for further targeted research on bioactive metabolites [19]. Previously, a total of 66 metabolites were definitely or tentatively identified from leaf samples and 44 metabolites from bark samples by untargeted metabolomics [20,21]. In total, it detected over 100 compounds by widely targeted metabolic profiling analysis [22]. This study conducted extensive targeted metabolomic analysis of extracts from mulberry roots, bark, leaves, fruits, and branches and identified 802 metabolites divided into 10 categories. This study elucidated the differences in metabolites among different medicinal parts. The reproducibility of each group of five biological samples was good. Previous research has mostly focused on the metabolite components of different varieties of leaves, different leaf times, and bark vs. leaf [23]. This study first systematically elucidated the distribution of metabolites and the differences in metabolites of four parts from *Morus alba* L. In addition, we combined chemical composition with TCM theory and used modern science to re-understand TCM.

MC is characterized by its sweet and cold nature and belongs to the lung meridian. It is effective in clearing the lungs, relieving asthma, and promoting diuresis to reduce edema. Modern medical research indicates that it can alleviate various diseases, such as cough, diabetes, inflammation, and cancer [24,25,26]. Among the herbs that belong to the lung meridian, terpenoid compounds are the most frequently occurring, with triterpenes being the most common; in addition, steroids, alkaloids, and flavonoids also have a higher frequency of occurrence in lung meridian herbs [27]. Mulberry bark is suitable for the treatment of symptoms of lung heat and cough. When treating lung cough and cough symptoms, Chinese medicines containing terpenoid compounds are often considered [28,29,30]. Correspondingly, our study found that the content of terpenoid components in mulberry bark was seven, eight, and ten times higher than that in mulberry leaves, mulberry fruit, and mulberry twigs, respectively. There is also literature indicating that among the terpenoid components in mulberry bark, the content of morroniside is the highest, and morroniside has been proven to possess various biological activities, such as anti-inflammatory and tissue repair promotion [31,32]. Additionally, terpenoids have the functions of decomposing fat, alleviating edema, and reducing mucus secretion in the respiratory tract, corresponding to the lung meridian of MCs [33].

MR has a slightly bitter taste and a neutral nature and belongs to the liver meridian. They exert significant effects in expelling wind dampness and benefiting the joints. Modern medical research indicates that mulberry twigs can alleviate diabetes, reduce cholesterol and blood sugar levels, enhance insulin sensitivity, and prevent liver damage, among other beneficial effects [34,35,36,37]. The highest content of adenosine in mulberry twigs plays a crucial role in various life processes, such as sleep and immune response. Abnormal adenosine signaling is often associated with conditions such as pain and neurodegenerative diseases, which correspond to the effects of mulberry twigs in expelling wind dampness and dredging the meridians [38].

MFo is sweet, bitter, and cold in nature and belongs to the lung and liver meridians. They are efficacious in dispersing wind heat, clearing the lungs to moisten dryness, and clearing the liver to improve eyesight. Modern medical research indicates that mulberry leaves have beneficial effects such as weight loss, anti-inflammatory, blood pressure improvement, antioxidation, antithrombogenesis, and antidiabetic properties [39,40,41,42]. The content of flavonoids in mulberry leaves is the highest, being 55 times, 7 times, and 37 times higher than that in mulberry bark, mulberry fruit, and mulberry twigs, respectively. The activities of flavonoid components are diverse, and have the effect of nourishing Yin, moistening dryness, cooling blood, and stopping bleeding [43,44,45,46].

MFr is sweet, sour, and cold in nature and belongs to the heart, liver, and kidney meridians. It possesses the effects of nourishing yin, enriching blood, promoting the production of body fluids, and moistening dryness. Modern medical research indicates that mulberry fruit can exert antioxidant effects and reduce blood lipid levels [47,48,49,50]. The total abundance of metabolites in MFr shows that its alkaloid content is the highest compound. Alkaloids can regulate metabolic reactions within the human body and exert whole-body effects. Among the alkaloids, the highest content is capsaicin, which has anti-inflammatory and antioxidant properties [51,52,53]. In combination with the polysaccharides of mulberry fruit [54], it corresponds to wasting thirst, benefits the five viscera and joints, promotes blood and qi circulation, calms the mind, sharpens intelligence, and keeps a person vigorous, clear, and youthful [55].

## 4. Materials and Methods

### 4.1. Plant Material

In May 2023, fresh fruit samples were harvested from 3-year-old trees in the *Morus alba* L. planting area of NANJING BOTANICAL GARDEN MEM.SUN YAT-SEN (32°03′ N, 118°50′ E), Nanjing, China, and identified by Professor Min Wei. These fruits were dehydrated in a drying oven at 40 °C and then ground into a powder. The plant was stored in a freezer at −80 °C. The MFo, MR, and MC were collected and dehydrated in May 2023.

### 4.2. Sample Preparation and Extraction

Grind the freeze-dried sample using a mixing mill at 60 Hz for 60 s. After adding 700 μL of extraction solution (methanol/water = 3:1, pre-cooled at −40 °C, containing internal standard), accurately weigh 50 mg aliquots of a single sample and transfer it to an Eppendorf tube. After vortexing for 30 s, homogenize the sample at 40 Hz for 4 min and sonicate in an ice water bath for 5 min. Repeat homogenization and ultrasonic treatment three times. Extract the sample overnight on a shaker at 4 °C, and then centrifuge at 12,000 rpm (RCF = 13,800 (× *g*), R = 8.6 cm) for 15 min at 4 °C. Carefully filter the supernatant through a 0.22 μm microporous membrane, then dilute the resulting supernatant 20 times with a methanol/water mixture, vortex for 30 s, transfer to a 2 mL glass bottle, take out 20 μL from each sample and combine them into QC samples. Store at −80 °C until analysis by UHPLC–MS.

### 4.3. UHPLC–MS Conditions

Use EXIONLC system (Sciex) for UHPLC separation. Mobile phase A is a 0.1% formic acid aqueous solution, and mobile phase B is acetonitrile. The column temperature is set to 40 °C. The temperature of the automatic sampler is set to 4 °C, and the injection volume is 2 μL. Sciex QTrap 6500+ (Sciex Technologies, Shanghai) is used for testing and development. Ion spray voltage: +5500/−4500 V. Curtain gas, ion source gas, and ion source gas were set at 35 psi, 1:60 psi, and 2:60 psi, respectively. The temperature was 400 °C, and DP was ±100 V. Sample measurements were performed with a gradient program that employed the starting conditions of 98% A, 2% B (Table 1).

### 4.4. Metabolite Identification and Quantification

SCIEX analysis workstation software (version 1.6.3) is used for MRM data acquisition and processing. Use an MS converter to convert MS raw data (.wiff) files to TXT format. The in-house R program and database are applied for peak detection and annotation. Commercial databases, including KEGG (http://www.kegg.jp/, accessed on 3 September 2024) and Metaboanalyst (http://www.metaboanalyst.ca/, accessed on 3 September 2024) was utilized to search for the pathways of metabolites.

### 4.5. Systematic Correlativity Analysis and Statistical Analysis

Pearson’s correlation, one-way ANOVA, and stratified (average linkage) clustering were performed for untargeted metabolic analysis. The *p*-value of the ANOVA was adjusted for the error detection rate.

## 5. Conclusions

In summary, the metabolites in different parts of *Morus alba* L. were analyzed using widely targeted metabolomics technology. A total of 802 metabolites were identified in the Mori Cortex, Mori Folium, Mori Fructus, and Mori Ramulus. Significant differences in the content of metabolites were observed among the different parts of the mulberry tree. This information is valuable for the food industry, medical treatment, and health benefits. The identification of key DAMs and related pathways provides valuable resources for future research, helping to better understand the physiology and metabolism of mulberry plants and reveal the characteristics of Traditional Chinese medicine, thus enhancing our understanding of its constituents and potential therapeutic benefits. This study marks the systematic synthesis of metabolites from different parts of the plant, providing a scientific basis for further research. These findings not only underscore the complexity of the mulberry’s metabolic landscape but also point to the need for targeted research to validate the biological activities of the identified DAMs. Future research should focus on validating the biological activities of the identified DAMs and elucidating the molecular mechanisms underlying their roles in the food industry and their health benefits.

## Figures and Tables

**Table 1:** Liquid chromatography mobile phase conditions ^1^.

Time	Flow Rate	A%	B%
0	400	98	2
0.5	400	98	2
10	400	50	50
11	400	5	95
13	400	5	95
13.1	400	98	2
15	400	98	2

^1^ The unit of time is min, and the flow rate unit is μL/min.

## Data Availability

Data will be made available upon request.

## Supplementary Materials

The following supporting information can be downloaded at: https://www.mdpi.com/article/10.3390/molecules29173981/s1, Table S1: The relative quantitative value of sample.
